# Transfusion-transmitted malaria: donor prevalence of parasitaemia and a survey of healthcare workers knowledge and practices in a district hospital in Ghana

**DOI:** 10.1186/s12936-016-1289-3

**Published:** 2016-04-23

**Authors:** Alex Owusu-Ofori, Dominic Gadzo, Imelda Bates

**Affiliations:** Department of Clinical Microbiology, Kwame Nkrumah University of Science and Technology, Kumasi, Ghana; College of Health Sciences, University of Kwazulu Natal University, Westville, South Africa; Akatsi South District Hospital, Akatsi, Ghana; Department of International Public Health, Liverpool School of Tropical Medicine, Liverpool, UK

**Keywords:** *Plasmodium falciparum*, Donor parasitaemia, Transfusion-transmitted malaria, Prevalence, Healthcare worker

## Abstract

**Background:**

Transfusion-transmitted malaria (TTM) is a risk of transfusion that has not been well described in malaria endemic regions. The risk of the recipient getting malaria is related to the prevalence of malaria in the blood donors. There is however little information on the prevalence of malaria among donors in Akatsi district of Ghana. Further, the knowledge and practices of healthcare workers to TTM is unknown. The study was undertaken to determine the prevalence of malaria parasite infection among blood donors and to evaluate the knowledge and practices of healthcare workers to TTM in the Akatsi district of Ghana.

**Methods:**

The study was conducted at Akatsi South District Hospital between May and August 2014. To screen for *Plasmodium falciparum*, 5 µl of capillary blood was obtained by finger prick from 200 participants (100 donors and 100 healthy controls). *Plasmodium falciparum* screening was done using CareStart™ Malaria Antigen kit. To obtain information regarding TTM knowledge and practices, questionnaires were completed by 100 health workers including nurses, doctors and laboratory staff.

**Results:**

The prevalence of *P. falciparum* was the same (10 %) in both donors and controls. All those who were malaria RDT positive were aged 15–25 years. Out of the 100 healthcare workers (31 males and 69 females) surveyed, 45 % of respondents (45/100) had never heard of transfusion-transmitted malaria. Almost all respondents (91 %) had not attended any lecture/seminar/workshop on blood transfusion in the past 12 months. There were 44 respondents (44 %) who wrongly said malaria was being screened for prior to transfusion in their hospital. However, 98.2 % (54/55) of those who had heard about TTM rightly stated that TTM can be prevented.

**Conclusion:**

The prevalence of *P. falciparum* parasitaemia is 10 % in healthy blood donors in the Akatsi district and represents a risk for TTM though the extent of this risk is unclear. Knowledge about TTM in healthcare workers in the district is low. Continuous education and in-service training may be a means to improve TTM knowledge and preventive practices by the health workers in the district.

## Background

The global burden of malaria has been considerably reduced over recent years [1]. However malaria still remains a major cause of morbidity and mortality [2]. In 2015, the estimated number of malaria cases in Africa was 218 million out of which were 395,000 deaths [3]. The gains made in malaria may in part be attributed to the coherent way in which scientists and public health workers have cooperated together. This has been done by developing integrated control and elimination programmes tailored for specific endemic settings and an active engagement in the research and development agenda required for malaria eradication [4].

Since the first reported case of transfusion-transmitted malaria (TTM) in 1911 [5], transfusion has been recognized as a well known source for malaria transmission. In non-endemic countries, TTM has been well described and can cause serious illness in non-immune recipients so measures have been put in place to prevent its occurrence. Potential blood donors who have visited malaria endemic regions are not allowed to donate blood for a specific period depending on the policy of that country and this can range from 6 months to several years. However in malaria endemic countries, the burden of TTM and its contribution to the overall burden of malaria is unknown. The prevalence of Plasmodium parasitaemia in blood donors varies depending on the endemicity of malaria in the region they live. In Africa, prevalence was found to range between 0.7–55 % [6]. These blood donors are a potential risk for TTM. From the few published studies on the incidence of TTM in malaria endemic countries it is clear that not all recipients of malaria-positive blood develop TTM and, because of the high endemicity some may develop malaria post-transfusion unrelated to the transfusion [7, 8].

Screening healthy donors for malaria in endemic countries poses a diagnostic challenge. This is because currently there is no screening test that is practical, affordable and suitably sensitive for blood banks [9]. Microscopy, which is the gold standard and is most frequently used, is not a sufficiently sensitive test especially when parasite density is low. Microscopy is time consuming and cannot realistically be used on a large scale as a screening tool in blood banks. Malaria rapid diagnostic tests (RDTs) are increasingly being used in malaria endemic settings and are useful for complying with the WHO strategy of confirming diagnosis before treatment. RDTS are simple to use, and may now be superior or equal in diagnostic sensitivity for uncomplicated *Plasmodium falciparum* malaria [10]. They have not been widely evaluated as a screening tool in blood donors.

The knowledge of health workers about TTM has not been previously evaluated. There is very little published data relating to practices and attitudes of health workers to TTM. A study found that practices in a teaching hospital are related to particular departments where doctors worked [11].

The aim of this study was to determine the prevalence of malaria in blood donors by RDT, compared to controls. In addition the knowledge, attitudes and practices of health workers in the district hospital in relation to transfusion malaria was determined.

## Methods

### Study site

The study was carried out in the Akatsi District Hospital (ADH), a public healthcare facility in the Akatsi South District of the Volta region of Ghana. ADH has staff strength of 140 healthcare workers including two doctors, a medical assistant, a pharmacist and a biomedical scientist. ADH is a 70-bed capacity, with a maternity unit, medical laboratory unit, family planning unit and an outpatient department. It also acts as referral centre for the surrounding primary health facilities in the district. Approximately 450 blood transfusions are carried out each year in ADH. Blood donations are from both voluntary and family replacement donors. The hospital blood bank routinely screens donor blood for hepatitis B, hepatitis C, HIV and syphilis.

### Enrolment

Blood donors and healthy controls from the community formed the study population to determine prevalence of malaria and health workers in ADH were the study population for the survey conducted. Donors who were aged between 15–55 years, had an haemoglobin (Hb) of more than 12.5 g/dL and were Hepatitis B, Hepatitis C, HIV, and syphilis negative were eligible for enrolment. These donors were consecutively enrolled after obtaining their written informed consent.

Healthy controls were also consecutively enrolled from individuals accompanying their relatives to the hospital, people working in the hospital or living in and around the hospital. Participants were excluded from the study if they show any sign of malaria, had fever or had been treated for malaria 4 weeks prior to enrolment. For the health worker survey, the study population was recruited from all health workers of ADH. A list of the names of all 142 health workers in ADH was obtained from the staff office. Workers were then approached individually to obtain their consent to participate in the study. Workers who gave their consent were given the study questionnaire to completely fill out. It was pretested and subsequently modified into a four-part document with a socio-demographics section, a knowledge section and a section on attitudes and practices. Responses to a total of 40 open and closed ended questions were anonymous so as to obtain frank answers without fear of victimization. The study questionnaire used was adapted and modified from a previously reported model [12].

Health workers were given 24 h to complete the questionnaire. Those who did not submit were reminded by phone or followed up in person. In total, 120 questionnaires were systematically distributed but 100 were returned for data entry and analysis.

### Malaria RDT testing

In total, blood samples were taken from 200 subjects, 100 blood donors and 100 controls.

Five microlitres of capillary blood sample was obtained from each donor and control participant for malaria parasite screening. Samples were collected at the start of the rainy season, between May and July 2014. This period represents the start of the malaria transmission season. Malaria testing was performed using CareStart™ Malaria Antigen test kit (Access Bio, Inc., USA); a histidine-rich protein (HRP-2) based rapid diagnostic tests (RDT) that detects *P. falciparum*. The use of this rapid kit has been previously been reported [13].

### Data analysis

Data collection was by paper case report sheets. Data were entered electronically and analysed using Statistical Package for Social Sciences (SPSS) for Windows (version 17.0). Prevalence of malaria, determined by malaria RDT positivity in donors and controls was calculated by dividing the number of positive results by the total number of samples.

### Ethical approval

Approval for this study was given by the Committee on Human Research Publication and Ethics of the School of Medical Sciences, Kwame Nkrumah University of Science and Technology in Kumasi, Ghana and the Management of Akatsi District Hospital in the Volta Region of Ghana. Study participants were assured of confidentiality and all data collected remained anonymous.

## Results

Donor and control groups were comparable in gender and age distribution. Males made up the majority in both groups; 55.0 % of donors and 57.0 % of controls respectively (Table 1). Those aged 15–25 years made up the majority and those above 46 years were the least in both groups (Table 1). The prevalence of *P. falciparum* was found to be 10 % in both donors and controls (Table 1). All those with positive malaria RDT malaria were aged 15–25 years. Males had a higher prevalence (10.9 %, 95 % CI 2.7–19.1 %) than females (8.9 %, 95 % CI 0.6–17.2 %) in donors. Similarly among controls, prevalence in males (14.0 %, 95 % CI 5.0–23.1 %) was higher than in females (4.7 %, 95 % CI 2.7–19.1 %) although the differences between males and females were not significant.

**Table 1:** Demographics and prevalence rate of Plasmodium prevalence rate in donors and non donors

Characteristics	Donors (N = 100) n (%)	Non-donors (N = 100) n (%)
Gender		
Males	55 (55.0)	57 (57.0)
Females	45 (45.0)	43 (43.0)
Age groups (years)		
15–25	79 (79.0)	55 (55.0)
26–35	12 (12.0)	35 (35.0)
36–45	8 (8.0)	7 (7.0)
>46	1 (1.0)	3 (7.0)
Prevalence rate	10 (10.0)	10 (10.0)

### Survey

The majority of the respondents to the questionnaire were females (69.0 %) and were aged between 20–30 years old (77.0 %). The most common job categories were nurses and nursing students followed by laboratory technicians (Table 2). Those who had worked less than 5 years made up 74.0 % of respondents and formed the majority. Ten percent of respondents had worked for more than 15 years in the hospital (Table 2).

**Table 2:** Socio-professional characteristics of participants in the health worker survey

Characteristics	Number (%)
Gender	
Male	31 (31.0)
Female	69 (69.0)
Age (years)	
20–30	77 (77.0)
31–40	11 (11.0)
41–50	5 (5.0)
51–60	7 (7.0)
Job category	
Nurse	63 (63.0)
Nursing student	16 (16.0)
Lab technician	6 (6.0)
Lab assistant	2 (2.0)
Doctors	2 (2.0)
Pharmacist technician	2 (2.0)
Pharmacists	1 (1.0)
Biomedical scientist	1 (1.0)
Others	7 (7.0)
Period of working life (years)	
<5	74 (74.0)
5–10	14 (14.0)
11–15	2 (2.0)
>15	10 (10.0)

### Health workers knowledge about TTM

Only 9.0 % of health workers had attended a seminar or workshop on blood transfusion in the past 12 months (Table 3). More than half, 55 out of 100 respondents (55.0 %) said they knew about TTM. The others had not heard of TTM. Of the 55 respondents who knew about TTM, 30 (54.5 %), the majority being nurses, regarded TTM as a major public health threat in Ghana (Table 3). Of the respondents that knew about TTM, 52/55 (94.5 %) thought that screening blood donors for malaria was necessary in Ghana. Regarding persons at risk of TTM in Ghana, although the majority, 27/55 of respondents (49.1 %) thought everyone was equally at risk for TTM, the rest thought that children under 5 years, pregnant women and people living with HIV/AIDS were particularly at-risk (Table 3).

**Table 3:** Health worker knowledge about transfusion-transmitted malaria

Question	Number of respondents	Answer	Number (%)
In the past 12 months have you attended a seminar/workshop on blood transfusion	100	Yes	9 (9.0)
		No	91 (91.0)
Have you heard of TTM?	100	Yes	55 (55.0)
		No	45 (45)
Is TTM a major public health threat in Ghana?	55	Yes	30 (54.5)
		No	11 (20.0)
		Not sure	14 (25.5)
Is malaria screening before blood donation necessary in Ghana?	55	Yes	52 (94.5)
		No	3 (5.5)
Who are the most at-risk group for TTM?	55	Everyone	27 (49.1)
		Children <5 years (A)	5 (9.1)
		Pregnant women (B)	2 (3.6)
		HIV/AIDS patients (C)	1 (1.8)
		A and B	5 (9.1)
		A and B and C	15 (27.3)
Can TTM be prevented?	55	Yes	54 (98.2)
		No	1 (1.8)
How can TTM be prevented?	55	Screening of donor blood	49 (89.1)
		Using mosquito nets	3 (5.5)
		Treating transfusion recipients	1 (1.8)
		Not sure	2 (3.6)
Do you agree with proposals for all transfusion recipient to be given anti-malarials	55	Yes	29 (52.7)
		No	26 (47.3)
Will routine use of anti-malarials lead to abuse and spread of drug resistance?	55	Yes	37 (67.3)
		No	18 (32.7)

There were 54 out of 55 respondents (98.2 %) who correctly knew that TTM is a preventable disease. Almost all respondents 89.1 % correctly mentioned screening of donated blood before donation as a prevention measure (Table 3). Only one out of 55 respondents (1.8 %) identified “treating transfusion recipients” as a prevention measure for TTM but 52.7 % agreed that transfusion recipients could be given anti-malarials as prophylaxis. The majority of respondents, 37 out of 55 (67 %) however agreed that routine use of anti-malarials in all transfusion recipients’ patients may lead to anti-malarial misuse and promote the spread of drug resistance (Table 3).

### Health workers’ attitudes and practices

Fifty percent of respondents (50/100) were directly involved with blood transfusion in Akatsi district hospital. Blood administration (41.0 %) and transfusion monitoring (30.0 %) were the most common activities while donor recruitment (3.0 %) and blood prescription (4.0 %) were the least common transfusion practices that staffs of ADH were involved in (Table 4).

**Table 4:** Responses by health workers on their attitudes and practices to transfusion-transmitted malaria

Question	Number of respondents	Answer	Number (%)
Are you directly involved in blood transfusion in your hospital?	100	Yes	50 (50.0)
		No	50 (50.0)
What do you do in relation to transfusion?	100	Prescribe blood	4 (4.0)
		Administer transfusion	41 (41.0)
		Monitor transfusion	30 (30.0)
		Screen blood	8 (8.0)
		Recruiting blood donors	3 (3.0)
		No response	14 (14.0)
Does your blood bank screen donor blood for malaria parasites?	100	Yes	44 (44.0)
		No	21 (21.0)
		Not sure	35 (35.0)
Will you transfuse malaria positive blood in case of an emergency?	55	Yes	30 (54.5)
		No	21 (38.2)
		Not sure	4 (7.3)
Will you transfuse syphilis positive blood in case of an emergency?	55	Yes	4 (7.3)
		No	51 (92.7)
Will you transfuse hepatitis B positive blood in case of an emergency?	55	Yes	0 (0)
		No	55 (100.0)
Giving routine anti-malarials	55	Yes, I will give everyone	16 (29.1)
		Yes, I will give only children	2 (3.6)
		No, I won’t give anyone	37 (67.3)

The majority of the respondents (44.0 %) wrongly stated that the hospital blood bank was screening for malaria parasite in blood donors before blood donation while a quarter of respondents were not sure if screening was being done or not (Table 4).

Among the 55 respondents who knew about TTM, 30 (54.5 %) indicated their willingness to transfuse malaria positive blood when faced with an emergency situation. In contrast, none of the 55 respondents were willing to transfuse hepatitis B positive blood in case of an emergency. The majority of the respondents 92.7 % (51/55) also indicated that they were unwilling to transfuse syphilis positive blood in a case of emergency (Table 4). Based on their current knowledge, 67.3 % of the workers (37/55) would not routinely give anti-malarials to transfusion recipients. A small proportion, 2/55 (3.6 %) would routinely give anti-malarials only to children receiving a transfusion, while 29.1 % would give anti-malarials routinely to every transfusion recipient (Table 4).

## Discussion

TTM in malaria endemic countries is attracting increasing attention with the current drive to reduce all avenues of transmission. TTM had been a neglected area of transfusion but since a meeting in Mombasa, Kenya identified TTM as a key gap in transfusion research in 2008 [14], there have been at least three major review articles and several other publications on different aspects of TTM.

This study showed that the prevalence of *P. falciparum* malaria in donors by RDT method was 10 %. This was similar to the findings from a study carried out in Kumasi, Ghana where *P. falciparum* prevalence was 13.7 % in donors by RDT [8]. Other African studies have found malaria prevalence in blood donors to be as high as 50 % [6]. The presence of parasitaemia in blood donors represents a potential risk to transfusion recipients and can result in TTM occurring in up to 28 % of recipients [7]. Certain factors such as refrigeration of blood at 4 °C and the immunity of the recipient may affect the susceptibility of individual recipients. Currently most blood banks in malaria-endemic African countries are not routinely screening blood for malaria. Ethical questions have been raised concerning whether it is appropriate to reject parasitaemic blood donors in malaria endemic countries where blood supplies are inadequate and the risks of clinically important TTM have not been well documented. Rejecting such donors will exacerbate already critical blood shortages and have the potential to increase mortality. Malaria RDTs may have a high rate of false positives test because of the presence of circulating antigens even after treatment. In recent times however, the role of malaria RDT testing cannot be disputed. RDT use has expanded greatly due to the WHO treatment guidelines which recommend the confirmation of parasitaemia prior to treatment with ACT [15]. The sensitivity of the RDTs has also been greatly improved and together with microscopy, they represent the best hope for accurate diagnosis as a key component of successful malaria control [16]. This study found the prevalence of *P. falciparum* malaria in donors and their controls to be the same (10 %). This is not surprising and is an indication that the blood donors are a true representation of the healthy adult population in the district. In many African countries the donor population is usually male dominated. However in this study, females were almost half of all donors. In this study, it is unclear why the prevalence of parasitaemia in males was more than double that of females in the control group. Some studies have established that parasitaemia is not significantly associated with the gender of donors. Ukaga et al. [17] found prevalence rates of 22.5 and 25.4 % for males and females respectively in Owerri, Nigeria.

The survey results showed that only 9 % of the respondents had attended any workshop on blood transfusion in the past 12 months. In Ghana, professional bodies require continuous professional development while health institutions encourage service training in all aspect of medicine. In many high-income countries, regular training on blood transfusion is mandatory, reflecting the importance of transfusion for patient safety and treatment. Much more advocacy is needed by the health professions and blood services in Ghana to ensure that transfusion is included in undergraduate curricula and in-service training programmes.

Knowledge about TTM was generally low among health workers in Akatsi. Almost half of the health workers had not heard about TTM. Malaria has been neglected a transfusion-transmitted infection, having been substantially overshadowed predominantly by a focus on HIV. Almost all respondents wrongly assumed that screening for malaria was being performed in Ghana. It is likely that the workers assumed that because routine screening takes place for HIV and hepatitis, malaria screening also takes place. It is unclear why a quarter of respondents admitted they were not sure if screening was done in their hospital but did not say so when questioned about screening across Ghana. If this level of misinformation occurs across Ghana then there is a clear need to provide education on TTM and particularly around strategies that can be used to reduce transmission through transfusion and to correct some assumptions that may negatively affect practice. It is interesting to observe that many health workers were unwilling to transfuse hepatitis B or syphilis positive blood but many were willing to transfuse malaria positive blood. Further investigations to determine the rationale for this will be useful.

Nearly all respondents (89.1 %) knew the most effective way of preventing TTM. This high level of knowledge could be possibly due to the effective malaria campaigns going on across the country. However, a third of health workers did not think that routine use of anti-malarials in transfusion will ultimately contribute to the spread of drug resistance. Current World Health Organization policies and treatment guidelines recommend that parasitological confirmation of malaria should be done before initiating the treatment [18]. However this advice is difficult to interpret in the context of blood transfusion and contradicts other guidelines [19] which advise that all transfusion recipients should receive anti-malarials. It is not surprising that this confusing guidance translates into lack of consistency in the prevention of TTM in clinical practice [11].

TTM is a ‘hidden’, neglected and under researched reservoir of ongoing malaria transmission. The recent announcement of a new one billion dollar fund to eliminate malaria means that renewed efforts are needed to understand and reduce the risks of malaria [20] transmission through blood transfusion.

## Conclusions

Healthy blood donors at the Akatsi district hospital have a 10 % prevalence rate of *P. falciparum* parasitaemia. This represents a potential risk for transmitting malaria via transfusion. While knowledge about TTM among healthcare workers in the district is low, continues education and in-service training may be a means to improve knowledge and preventive practices by the health workers in the district.

## Abbreviations

TTM: transfusion-transmitted malaria
RDT: rapid diagnostic test
ADH: Akatsi District Hospital
